# Dietary Oryzanol (Ory) Improved the Survival and Growth of Large Yellow Croaker (*Larimichthys crocea*) Larvae via Promoting Activities of Digestive Enzymes, Antioxidant Capacity, and Lipid Metabolism

**DOI:** 10.1155/2024/8368883

**Published:** 2024-05-14

**Authors:** Wenxuan Xu, Yuntao Wang, Jianmin Zhang, Jiahui Liu, Yongtao Liu, Wenxing Huang, Chuanwei Yao, Kangsen Mai, Qinghui Ai

**Affiliations:** ^1^Key Laboratory of Aquaculture Nutrition and Feed (Ministry of Agriculture), Key Laboratory of Mariculture (Ministry of Education), Ocean University of China, Qingdao, Shandong, China; ^2^Laboratory for Marine Fisheries Science and Food Production Processes, Qingdao National Laboratory for Marine Science and Technology, 1 Wenhai Road, Qingdao 266237, Shandong, China

## Abstract

A feeding study lasting 30 days was carried out to determine the effects of dietary Oryzanol (Ory) on the survival, growth, antioxidant capacity, peptic enzymes, as well as lipid metabolism of *Larimichthys crocea* larvae (11.87 ± 0.59 mg) using four different Ory concentrations in microfeed formulations (0, 20, 40, and 80 mg/kg), all preserving isolipidic (18.25% crude lipid) and isonitrogenous (52.08% crude protein) conditions. Results revealed that larvae given 40 and 80 mg/kg Ory revealed markedly higher survival rates; in particular, the 80 mg/kg Ory larvae exhibited a considerably higher specific growth rate than the control. Furthermore, the 80 mg/kg dietary Ory resulted in increased peptic enzyme activity, indicating heightened digestive capabilities of larvae. Meanwhile, Ory supplementation at 20, 40, and 80 mg/kg also increased antioxidant enzyme activities and reduced malondialdehyde levels, indicating an enhanced antioxidant capacity. Moreover, the incorporation of 20 and 40 mg/kg Ory demonstrated efficacy in enhancing the lipid metabolism of the larvae. This was evident in the reduction of triglyceride and total cholesterol levels in the larval visceral mass, attributed to the downregulation of genes that participate in lipid synthesis, and the upregulation of genes associated with lipid catabolism. Overall, the study suggests that the addition of Ory (ranging from 40 to 80 mg/kg) significantly improves both survival and developmental aspects, possibly mediated by enhanced digestion and antioxidative effects, alongside the induction of lipid metabolism in *Larimichthys crocea* larvae.

## 1. Introduction

The large yellow croaker (*Larimichthys crocea*), esteemed as a prominent foremost marine fishery species in China, primarily inhabits coastal regions and estuaries in the northwest Pacific [1–3]. Nevertheless, ongoing problems are hindering the development of the large yellow croaker sector, notably suboptimal survival rates and inadequate developmental progress among large yellow croaker larvae, acting as primary constraints in larviculture [4, 5]. Addressing these issues has been a focal point for numerous researchers, prompting extensive efforts to devise effective methodologies.

The integration of plant extracts into artificial micro diets has shown promise in enhancing the viability and development of fish larvae [6–8]. Among different plant extracts, Oryzanol (Ory), which occurs naturally in crude rice (*Oryza Sativa*) bran oil at concentrations of 1.5%–3%, is particularly notable [9]. Ory consists of a blend of at least 10 ferulic acid esters, among which 24-methylene cycloartenyl ferulate has been recognized as the principal constituent, constituting approximately 80% of its composition [9]. Previous research in mammals and livestock has highlighted the beneficial role of Ory in terms of survival and growth [10–12]. Besides, Akiyama et al. [13] reported Ory's high radical scavenging capacity in mammalian systems. Moreover, studies by Wang et al. [10] and Minatel et al. [14] demonstrated the Ory is associated with anti-inflammatory properties and cholesterol-lowering effects in mammals and livestock, respectively. Moreover, acknowledged as a nutritional component for fish, the main way that Ory's beneficial effects were confirmed was by suppressing oxidative stress in red sea bream (*Pagrus major*) [15].

As previously emphasized, the quest for substances capable of augmenting the performance is one of the strategy in the aquaculture of larval culture. However, the potential impact of Ory on the development and well-being of large yellow croaker larvae remains unexplored. Consequently, the current study looked at how dietary supplementation of Ory affected important factors in large yellow croaker larvae, including peptic enzyme activity, developmental markers, survival rates, and antioxidant capacity together with lipid metabolism.

## 2. Materials and Methods

### 2.1. Ingredients and Diets Formulation of Fish Feed

Four microdiets were devised to maintain consistent levels of energy (18.25% crude lipid) and nitrogen (52.08% crude protein), with different Ory concentrations at 0, 20, 40, and 80 mg/kg dry feed, as presented in Table 1. The Ory utilized in this study was provided by Shanghai Aladdin Biochemical Technology Co., Ltd., China, ensuring a purity exceeding 99.0%.

The feed constituents underwent an ultrafine grinding process and were sieved through a 100 *μ*m screen. Following a prescribed formula, these ingredients were gradually introduced into the mixer and continuously mixed for 25 min. With a single screw spheronization apparatus and spherical squeezer, extrusion-spheronization approach was applied to produce hard pellet microdiets with a diameter of 2.5 mm. To attain a moisture content below 10%, all pellets underwent baking for 8–12 hr at 50°C until a constant weight was achieved.

The resultant feeds were subsequently squeezed and sifted to obtain microfeed with special dimensions ranging from 250 to 380 *μ*m as well as 380–500 *μ*m, respectively. These pellets were individually packed into ziplock bags and stored at −20°C until needed. During the phase, fish larvae were fed pellets sized between 250 and 380 *μ*m from 15 to 25 days after hatch (DAH), while pellets sized between 380 and 500 *μ*m were administered to the larvae from 26 to 45 DAH.

### 2.2. Experimental Procedures

Same batch of large yellow croaker larvae originating from the Marine and Fishery Science and Technology Innovation Base in Zhejiang, China, was meticulously chosen and raised under controlled conditions. The criteria for selection were homogeneity, disease-free, with a mean body weight of 11.87 ± 0.59 mg. From 3 to 8 days after birth, larvae fed exclusively on rotifers (*Brachionus plicatilis*) at a concentration ranging from 0.5 × 10^4^ to 1.5 × 10^4^ individuals per liter. Subsequently, from 6 to 11 DAH, the diet transitioned to brine shrimp (*Artemia nauplii*) at a density from 1.0 × 10^3^ to 1.5 × 10^3^ individuals per liter. Between 10 and 14 days of age, larvae were fed live copepods (*Calanus sinicus*) along with an experimental diet of 0 mg/kg Ory. Thereafter, the trial diet was the only food given to the larvae. Twelve white plastic tanks, each 220 L in volume and with a density of 3,000 larvae, were used to carry out the test. These tanks were randomly partitioned into three groups, with each replicate group corresponding to a distinct experimental feed. Feeding of the larvae occurred manually seven times daily (at 06:30, 09:30, 12:30, 15:30, 18:30, 21:30, and 0:30) from 15 to 45 DAH to meet the requirements of larvae. The aquatic environment was carefully maintained with water parameters set at 24.5 ± 1.5°C, salinity maintained at 23 ± 2 g/L, and a pH of 8.0 ± 0.2. Water quality was sustained by sieving and renewing the water by 150%–200% daily.

### 2.3. Sampling and Dissection

Considering the fragility of larvae and high risk of stress death, 90 larvae (15 DAH) were randomly chosen from the whole larvae population for initial measurements of original body length (IBL) and body weight (IBW) in order to minimize the stress caused by manipulation on larvae. Toward the conclusion of the experiment, the survival rate (SR) was surveyed by assessing the left larvae in each tank. To ensure an accurate assessment, larvae at 45 DAH underwent a fasting period the day prior to sampling. Subsequently, in order to determine the final body weight (FBW) and final body length (FBL), 50 individuals were randomly selected from each tank.

For gene expression analysis, 20 larvae were chosen at random from each tank, dissected on ice, and later separated to remove visceral tissues, which included the gut, liver, heart, and spleen. The tissues that had been dissected were immediately stored in 2 mL cryogenic vials devoid of RNase and quickly submerged in liquid nitrogen to preserve their integrity. Additionally, to assess digestive enzyme activities, 50 larvae were meticulously dissected under a low-temperature cutting microscope, extracting PS and IS following the anatomical guidelines outlined by Cahu and Infante [16].

### 2.4. Body Composition Analysis

The remaining larvae from each tank, subsequent to the sampling procedure, were utilized to determine their body composition. Through drying the specimens in a ventilated oven at 105°C until a steady weight was reached, the moisture content of the material was determined. The quantification of crude lipid and crude protein content within the specimens adhered to the procedures outlined by the Association of Official Analytical Chemists [17]. The content of crude protein involved the Kjeldahl method (KjeltecTM 8400, FOSS, Tecator, Sweden) was calculated as the nitrogen content multiplied by the factor 6.25, while the content of crude lipid was estimated via the Soxhlet method for ether extraction (B-801, Switzerland). These analytical procedures were replicated for each group of specimen to ensure accuracy and reliability of the measurements.

### 2.5. Digestive Enzyme Activities Assay

Individual specimens of IS and PS weighing between 0.2 and 0.3 g were homogenized in phosphate-buffered saline (2 mL) at 0°C (pH = 7.4) and centrifuged for 10 min at 3,300 *g*. After centrifugation, the supernatant was gathered for further examination. Cleared brush border membrane (BBM) was separated from intestinal segment homogenates following the method outlined by Crane et al. [18].

Assessment of changes in trypsin activity was conducted utilizing Na-Benzoyl-DL-arginine-p-nitroanilide (BAPNA, B-4875, Sigma, USA) as substrates, following the procedures described by Holm et al. [19]. The determination of leucine-aminopeptidase (LAP) activity was carried out following the methodology established by Maroux et al. [20]. Test kits encompassing total protein, *α*-amylase, lipase, and alkaline phosphatase (AKP) were procured from Nanjing Jiancheng Bio-Engineering Institute, China. All assays were meticulously executed adhering strictly to the specified sequence and protocols.

### 2.6. Antioxidant Activities and Malondialdehyde (MDA), Triglyceride (TG), and Total Cholesterolcontent (T-CHO) Content Assay

The underwent measurement of the weight of larval visceral mass, followed by homogenization within a phosphate-buffered saline solution (0°C, pH = 7.4) using a tissue-to-saline ratio of 1 : 9 (g:mL). After homogenization, the visceral mass homogenate underwent centrifugation for 10 min at 3,300 g. The resulting supernatant was employed to assess the content of malondialdehyde (MDA) and the antioxidant enzyme activities. The evaluation of catalase (CAT), total antioxidant capacity (T-AOC), total superoxide dismutase (T-SOD), and content of malondialdehyde (MDA), total cholesterolcontent (T-CHO), as well as the content of triglyceride (TG), involved the use of commercially available assay kits procured from Nanjing Jiancheng Bio-Engineering Institute, China. All experimental procedures were meticulously executed obey the prescribed protocols strictly, to ensure precision and reliability of the results.

### 2.7. Extraction of RNA and Real-Time Quantitative PCR

Liquid nitrogen was utilized to pulverize the samples, which were subsequently treated with Trizol reagent (Takara, Japan). Subsequent RNA extraction procedures adhered strictly to the protocols provided by the manufacturer. The quality of the isolated RNA was assessed through electrophoresis, while the concentration of total RNA was identified through the NanoDrop® 2000 spectrophotometer (Thermo Fisher Scientific, USA). To eradicate DNA contamination within the RNA samples, RNase-Free DNase (Takara, Japan) was employed. Following decontamination, the RNA was reversely transcribed into complementary DNA (cDNA) with the Prime Script-RT reagent Kit (Takara, Japan).

Polymerase chain reaction (PCR) amplification was implemented through a thermal cycler (CFX96TM Real-Time System, Bio-Rad, USA). Primers targeting diacylglycerol acyltransferase 2 (*dgat2*), fatty acid synthase (*fas*), stearoyl-coenzyme A desaturase 1 (*scd1*), acyl-CoA oxidase (*aco*), cluster of differentiation 36 (*cd36*), sterol-regulatory factor relative protein 1 (*srebp1*), peroxisome proliferators-activated receptor *α* (*pparα*), carnitine palmitoyl transferase-1 (*cpt-1*), *β*-actin, and hepatic lipase (*hl*) were designed according to published sequences from Zuo et al. [21], Xu et al. [22], Yan et al. [23], and Cai et al. [24] (Table 2). The PCR's thermal profile comprised an initial denaturation at 95°C for 2 min, and then 39 cycles of denaturation at a temperature of 10 s each, 95°C and extension at 20 s, 72°C. The fluorescence signals obtained in the stage of amplification were normalized to the expression level of *β*-actin utilizing the 2^−*ΔΔ*CT^ approach proposed via Livak and Schmittgen [25].

### 2.8. Calculations and Statistical Analysis

The following formulas were applied.

1. Survival rate (SR, %) was calculated utilizing the following formula:

(1) $$\begin{array}{c} \text{SR }\left(\%\right)=100\times N_{\text{t}}/N_{0}, \end{array}$$

where *N*_t_ represents the final larvae count in each tank at the conclusion of the observation period and *N*_0_ is the larval initial count in each tank at the beginning of the observation period.

(2) Specific growth rate (SGR, % per day) was determined through the following equation:

(2) $$\begin{array}{c} \text{SGR }\left(\%/\text{day}\right)=100\times \left(\ln W_{\text{t}}-\ln W_{\text{0}}\right)/d, \end{array}$$

where *W*_t_ and *W*_0_ denote the initial and final wet body weight of per larval in grams, respectively, and *d* denotes the duration of the test (days).

These calculations allowed for the quantitative assessment of growth performance and survival efficiency of the larvae over the specified trial period.

All data underwent statistical analysis with SPSS Statistics 25.0 software (SPSS Inc., USA). Initially, it was subjected to ANOVA to determine if there was a significant difference between the groups, Tukey's post hoc tests were subsequently conducted to further elucidate the differences. The threshold of statistical significance was *P* < 0.05. The results of the analyses are presented as means, with standard error of the mean (SEM) provided for each, denoted as mean ± SEM.

## 3. Results

### 3.1. Survival, Growth Performance, and Body Composition

Results revealed that larvae receiving 40 and 80 mg/kg of Ory exhibited a distinctly increase in larval SR in comparison with the control group (*P* < 0.05) (Table 3). Additionally, supplementation with 80 mg/kg of Ory markedly enhanced the larval FBW and SGR in comparison with those in the control (*P* < 0.05) (Table 3). There were no discernible changes in FBL and body composition among the various treatment groups for the larvae (*P* > 0.05) (Tables 3 and 4).

### 3.2. Activities of Digestive Enzymes

In comparison to the control group, the supplementation of 80 mg/kg of Ory substantially elevated the trypsin activities in both IS and PS of large yellow croaker larvae (*P* < 0.05) (Table 5). Additionally, the supplementation of Ory at concentrations of 20, 40, and 80 mg/kg was associated with a significant increase in the LAP and AKP activities within the BBM of the larvae (*P* < 0.05) (Table 5). The lipase and larval *α*-amylase activities did not exhibit significant variations (*P* > 0.05) (Table 5).

### 3.3. Activities of Antioxidant Enzymes and MDA Content

**Figure 1:**
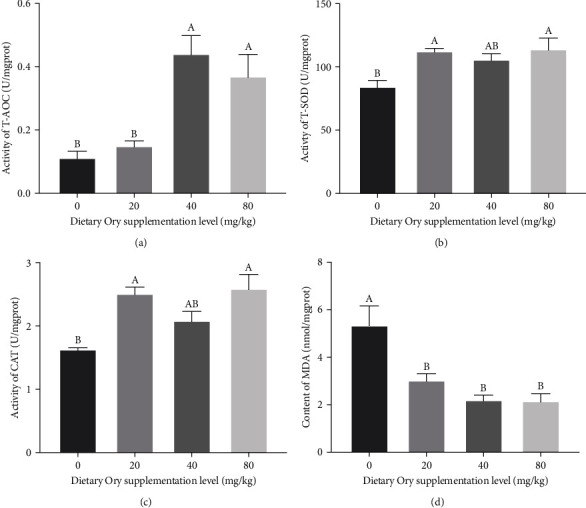
Effects of supplementation of Oryzanol on antioxidant capability in visceral mass of large yellow croaker larvae. (a) T-AOC, total antioxidant capacity; (b) T-SOD, total superoxide dismutase; (c) CAT, catalase; (d) MDA, malondialdehyde. Values are means (*n* = 3), with their standard errors represented by vertical bars. Bars bearing the same letters were not significantly different (*P* > 0.05, one-way ANOVA with post-hoc Tukey HSD test).

In comparison to the control group, the T-AOC activity of larvae fed 40 and 80 mg/kg of Ory was noticeably increased (*P* < 0.05) (Figure 1(a)). Furthermore, the addition of Ory to the food at 20 and 80 mg/kg caused a significant rise in the larval CAT and T-SOD activity (*P* < 0.05) (Figures 1(b) and 1(c)). Simultaneously, there was an evident decrease in the MDA concentration when Ory was added at 20, 40, and 80 mg/kg (*P* < 0.05) (Figure 1(d)).

### 3.4. Triglyceride (TG), Total Cholesterolcontent (T-CHO) Contents, and the mRNA Expression of Genes Associated with Lipid Metabolism

**Figure 2:**
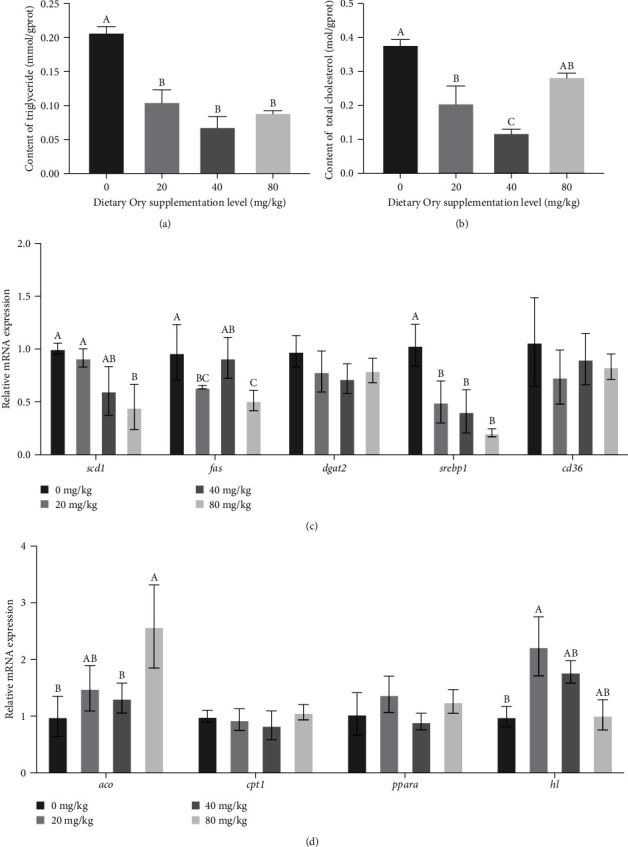
Effects of supplementation of Oryzanol on triglyceride content, total cholesterol, and relative mRNA expression of genes involved in lipid metabolism in visceral mass of large yellow croaker larvae. (a) The triglyceride content; (b) the total cholesterol content; (c) relative mRNA expression of genes involved in lipogenesis; (d) the relative mRNA expression of genes involved in lipid consumption. Values are means (*n* = 3), with their standard errors represented by vertical bars. Bars bearing the same letters were not significantly different (*P* > 0.05, one-way ANOVA with post-hoc Tukey HSD test).

The results revealed a marked reduction in triglyceride (TG) content in larvae which was markedly reduced with the inclusion of 20, 40, and 80 mg/kg of Ory in contrast to the control (*P* < 0.05) (Figure 2(a)). Additionally, supplementation with 20 and 40 mg/kg of Ory dramatically reduced the amount of T-CHO in large yellow croaker larvae (*P* < 0.05) (Figure 2(b)). Notably, the genes mRNA expression related to lipid synthesis—*srebp1*, *fas*, and *scd1*—was evidently downregulated via supplementation of Ory (*P* < 0.05) (Figure 2(c)). Compared with the control group, the *scd1* mRNA expression was remarkably downregulated with 80 mg/kg of Ory (*P* < 0.05), while expression of *fas* was evidently decreased by 20 and 80 mg/kg of Ory (*P* < 0.05). Moreover, supplementation with 20, 40, and 80 mg/kg of Ory resulted in a remarkable reduction of mRNA expression of *srebp1* in larvae (*P* < 0.05) (Figure 2(c)). Meanwhile, genes associated with lipid catabolism, *aco* and *hl*, exhibited significant upregulation following Ory supplementation (Figure 2(d)). Specifically, the mRNA expression of *aco* was markedly increased with 80 mg/kg Ory addition, while *hl* expression significantly rose by the 20 mg/kg Ory inclusion (*P* < 0.05) (Figure 2(d)).

## 4. Discussion

Results indicated that larvae supplemented with 40 and 80 mg/kg of Ory exhibited notably higher SR compared to other groups. Moreover, the inclusion of 80 mg/kg of Ory significantly enhanced both the FBW and SGR of the larvae. These findings suggest that the supplementation of 40 and 80 mg/kg of Ory could potentially contribute to the larval enhanced survive and development. A parallel conclusion was drawn by Nagasaka et al. [26], where the administration of dietary 10 mg/kg Ory for 2 months notably improved the body weight of rainbow trout (*Oncorhynchus mykiss*) juveniles. Previous conclusions have consistently highlighted the benefit of Ory in improving the survival and development of fish, potentially associated with its effects on digestion, antioxidant capacity, and lipid metabolism [27].

Early and pronounced development of digestive enzymes has been linked to the increased development and survival of marine fish larvae after weaning, as highlighted in studies by Cara et al. [28] and Imentai et al. [29]. Additionally, the efficacy of exogenous botany extracts in stimulating the maturation of peptic enzymes in marine fish larvae has been documented by researchers, as issued in the works of Huang et al. [30] and Li et al. [31]. Within the ambit of this investigation, the enhancement of trypsin activities in PS and IS was significantly observed with the addition of 20, 40, and 80 mg/kg of Ory. This outcome suggests that dietary supplementation of Ory could improve protein digestion in fish larvae. Moreover, notable improvements were observed in the AKP and LAP activities within the BBM. LAP and AKP are predominantly located in the BBM of the intestinal tract and serve as indicators of intestinal digestion and absorption functionality [32]. AKP, in particular, is essential for the hydrolysis of dietary phospholipids [33], a process critical for lipid digestion [34]. The outcomes of this research underscore the significant enhancement in digestive enzyme activities conferred by Ory supplementation in large yellow croaker larvae, positing this investigation as a pioneering study on the effect of Ory on the digestive efficiency within the diets of marine fish larvae.

Immature antioxidant function in marine fish larvae renders them impressionable to oxidative stress, which often results in diminished survival and growth rates [35]. Notably, via raising the activity of antioxidant enzymes, Ory has been found to be a mitigating agent against oxidative stress [36, 37]. This research elucidates that dietary inclusion of Ory significantly bolstered the antioxidant defense in large yellow croaker larvae, evidenced by elevated activities of key enzymes for instance T-SOD, T-AOC, CAT, and alongside a decrease in the levels of (MDA). These findings parallel the research conducted by Maoka et al. [15], which highlighted the positive impact of a 0.5% dietary supplementation of Ory on the antioxidant capacity of red sea bream (*Pagrus major*) [15]. In essence, the integration of 40 and 80 mg/kg of Ory into the diet demonstrated a remarkable ability to benefit the antioxidant defense mechanism of large yellow croaker larvae.

The substantial energy expenditure in fish larvae underscores the significance of enhancing lipid metabolism, as it can yield more energy to facilitate protein synthesis and foster the larval overall development [5, 38]. In line with these observations, the supplementation of 20, 40, and 80 mg/kg of Ory markedly reduced the TG content in the visceral mass of the larvae, while 20 and 40 mg/kg of Ory notably substantially diminished the T-CHO content, signifying the lipid-lowering effect of Ory in fish larvae. Crucial genes in the lipid cell-generating process, such as *scd1*, *fas*, and *dgat2*, are pivotal for lipid metabolism [39, 40]. The current project observed a significant decrease in the *dgat2*, *fas*, and *scd1* mRNA expressions, suggesting that Ory had a moderate suppressive effect on larval lipid production. Concurrently, Ory supplementation led to the upregulation of genes associated with lipolysis, such as *aco* and *hl*, which activate fatty acid oxidation and expedite the process of lipid expenditure. This outcome aligns with the findings of Ma et al. [41] in mice. Collectively, these findings underscore Ory's substantial effect in augmenting lipid metabolism within large yellow croaker larvae, underscoring its potential utility in aquaculture nutrition strategies.

## 5. Conclusions

In conclusion, this study confirmed that adding Ory to a diet in the right amounts produces beneficial effects on the development of digestion, antioxidant capabilities, and lipid metabolism, ultimately enhancing the survival and developmental aspects of large yellow croaker larvae.

This research introduces a novel perspective for advancing larval culture practices within the industry.

Based on the outcomes of the current experiment, the most suitable dosage range of Ory incorporated into the feed for large yellow croaker larvae falls within 40–80 mg/kg, among the evaluated doses of 20, 40, and 80 mg/kg.

## Figures and Tables

**Table 1:** Formulation and proximate analysis of the experimental diets (percentage dry matter).

Ingredient percentage dry diet	Diets (Oryzanol, mg/kg)			
	Diet1 (0)	Diet2 (20)	Diet3 (40)	Diet4 (80)
White fish meal^a^	45	45	45	45
Krill meal^b^	22	22	22	22
Squid viscera meal^a^	3	3	3	3
Yeast hydrolysate	3.5	3.5	3.5	3.5
Strong flour	5.0	5.0	5.0	5.0
*α*-Starch	2.6	2.6	2.6	2.6
Sodium alginate	2	2	2	2
Vitamin premix^c^	1.5	1.5	1.5	1.5
Mineral premix^d^	1.3	1.3	1.3	1.3
Ascorbyl polyphosphate	0.2	0.2	0.2	0.2
Calcium bis	2	2	2	2
Mould inhibitor	0.05	0.05	0.05	0.05
Antioxidant	0.05	0.05	0.05	0.05
Choline chloride	0.2	0.2	0.2	0.2
Fish oil	6.5	6.5	6.5	6.5
Soybean phospholipids	5.0	5.0	5.0	5.0
Microcrystalline Cellulose	0.1	0.098	0.096	0.092
Oryzanol^e^	0	0.002	0.004	0.008
Analyzed nutrients composition (dry matter basis)				
Crude protein (%)	52.10	51.98	52.37	51.87
Crude lipid (%)	18.49	18.20	18.28	18.03

^a^Commercially available from Great Seven Biotechnology Co., Ltd. in Shandong, China; elementary composition (dry matter): White fish meal, crude protein, 71.73%, crude lipid, 4.76%; Squid viscera meal, crude protein, 39.01%, crude lipid, 11.34%. ^b^Commercially available from Beijing Huaxia Houde Co., Ltd. (Beijing, China). Krill meal, crude protein, 64.86%, crude lipid, 8.0%. ^c^Composition of vitamin premix (IU or g/kg): vitamin A palmitate, 3,000,000 IU; vitamin D_3_, 1,200,000 IU; DL-*α*-vitamin E, 40.0 g/kg; menadione, 8.0 g/kg; thiamine-HCl, 5.0 g/kg; riboflavin, 5.0 g/kg; D, calcium pantothenate; 16.0 mg/kg; pyridoxine-HCl, 4.0 mg/kg; inositol, 200.0 mg/kg; biotin, 8.0 mg/kg; folic acid, 1.5 mg/kg; 4-aminobenzoic acid, 5.0 mg/kg; niacin, 20.0 mg/kg; vitamin B_12_, 0.01 mg/kg; L- ascorgyl-2-monophosphate-Na (3%), 2,000.0 mg/kg. ^d^Composition of mineral premix (g/kg premix): Ca(H_2_PO_4_)·H_2_O, 675.0; C_O_SO_4_·H_2_O, 0.15; CuSO_4_ · H_2_O, 5.0; FeSO_4_ · 7H_2_O, 50.0; KCl, 0.1; MgSO_4_ · 2H_2_O, 101.7; MnSO_4_ · 2H_2_O, 18.0; NaCl, 80.0; NaSeO_3_·H_2_O, 0.05; ZnSO_4_ · 7H_2_O, 20.0. ^e^The Oryzanol was purchased from Shanghai Aladdin Biochemical Technology Co., Ltd., in China. The purity was above 99.0%.

**Table 2:** Primers used for quantitative PCR.

Gene	Forward (5′−3′)	Reverse (5′−3′)	Accession number
*scd1*	AAAGGACGCAAGCTGGAACT	CTGGGACGAAGTACGACACC	Xu et al. [22]
*fas*	CAGCCACAGTGAGGTCATCC	TGAGGACATTGAGCCAGACAC	Cai et al. [24]
*dgat2*	TTCGGTGCTTTCTGCAACTTCG	AAGGATGGGGAAGCGGAAGT	Yan et al. [23]
*srebp1*	TCTCCTTGCAGTCTGAGCCAAC	TGAGGACATTGAGCCAGACAC	Cai et al. [24]
*cd36*	GAGCATGATGGAAAATGGTTCAAAG	CTCCAGAAACTCCCTTTCACCTTAG	Cai et al. [24]
*aco*	AGTGCCCAGATGATCTTGAAGC	CTGCCAGAGGTAACCATTTCCT	Yan et al. [23]
*cpt-1*	GCTGAGCCTGGTGAAGATGTTC	TCCATTTGGTTGAATTGTTTACTGTCC	Yan et al. [23]
*pparα*	GTCAAGCAGATCCACGAAGCC	TGGTCTTTCCAGTGAGTATGAGCC	Zuo et al. [21]
*hl*	TCCGTCCATCTATTCATTGACTCTC	GCCACTGTGAACCTTCTTGATATTG	Cai et al. [24]
*β-actin*	GACCTGACAGACTACCTCATG	AGTTGAAGGTGGTCTCGTGGA	Yan et al. [23]

*Abbreviations*. *scd1*, stearoyl-CoA desaturase 1; *fas*, fatty acid synthase; *dgat2*, diacylgycerol acyltransferase 2; *srebp1*, sterol-regulatory element binding protein 1; *cd36*, cluster of differentiation 36; *aco*, acyl-CoA oxidase; *cpt-1*, carnitine palmitoyl transferase-1; *pparα*, peroxisome proliferators-activated receptor *α*; and *hl*, hepatic lipase.

**Table 3:** Effects of supplementation of Oryzanol on survival and growth performance of large yellow croaker larvae (means ± SEM, *n* = 3)^1^.

Parameters	Diets (Ory supplementation level, mg/kg)			
	Diet1 (0)	Diet2 (20)	Diet3 (40)	Diet4 (80)
Initial body length (IBL, mm)	6.23 ± 0.98	6.23 ± 0.98	6.23 ± 0.98	6.23 ± 0.98
Initial body weight (IBW, mg)	11.87 ± 0.59	11.87 ± 0.59	11.87 ± 0.59	11.87 ± 0.59
Final body length (FBL, mm)	21.25 ± 0.48	21.90 ± 0.06	21.22 ± 0.46	21.97 ± 0.50
Final body weight (FBW, mg)	171.33 ± 14.44^b^	193.67 ± 7.86^ab^	192.00 ± 7.00^ab^	223.67 ± 6.36^a^
Specific growth rate(SGR %/day)	8.88 ± 0.27^b^	9.30 ± 0.13^ab^	9.27 ± 0.12^ab^	9.78 ± 0.96^a^
Survival rate(SR %)	14.31 ± 2.02^b^	20.73 ± 1.00^ab^	27.64 ± 3.25^a^	25.56 ± 2.73^a^

^1^Data are presented as means ± SEM. Means in each row sharing the same superscript letter or absence of superscripts are not significantly different decided by one-way ANOVA with post-hoc Tukey HSD test (*P*  > 0.05). SEM, standard error of means.

**Table 4:** Effects of supplementation of Oryzanol on body composition in large yellow croaker larvae (means ± SEM, *n* = 3)^1^.

Parameters	Diets (Ory supplementation level, mg/kg)			
	Diet1 (0)	Diet2 (20)	Diet3 (40)	Diet4 (80)
Crude protein (%)	57.38 ± 1.53	57.72 ± 1.09	58.46 ± 0.24	59.01 ± 1.00
Crude lipid (%)	22.86 ± 1.72	22.72 ± 2.06	21.03 ± 0.98	20.58 ± 1.24
Moisture (%)	83.47 ± 1.00	82.23 ± 2.36	84.10 ± 2.02	84.72 ± 3.31

^1^Data are presented as means ± SEM. Means in each row sharing the same superscript letter or absence of superscripts are not significantly different decided by one-way ANOVA with post-hoc Tukey HSD Test (*P*  > 0.05). SEM, standard error of means.

**Table 5:** Effects of supplementation of Oryzanol on activities of main digestive enzymes of large yellow croaker larvae (means ± SEM, *n* = 3)^1^.

Parameters	Diets (Ory supplementation level, mg/kg)			
	Diet1 (0)	Diet2 (20)	Diet3 (40)	Diet4 (80)
Amylase (U/mg·protein)				
PS	0.24 ± 0.06	0.17 ± 0.02	0.27 ± 0.04	0.28 ± 0.08
IS	0.65 ± 0.09	0.58 ± 0.09	0.57 ± 0.10	0.57 ± 0.06
Trypsin (U/mg·protein)				
PS	0.33 ± 0.04^b^	0.37 ± 0.01^ab^	0.38 ± 0.07^ab^	0.54 ± 0.03^a^
IS	0.28 ± 0.03^b^	0.38 ± 0.03^ab^	0.46 ± 0.06^ab^	0.61 ± 0.08^a^
Try-IS/ (PS + IS)	0.50 ± 0.04	0.51 ± 0.03	0.55 ± 0.06	0.53 ± 0.06
Lipase (mU/mg·protein)				
PS	0.49 ± 0.08	0.60 ± 0.14	0.50 ± 0.04	0.54 ± 0.03
IS	0.33 ± 0.06	0.44 ± 0.04	0.41 ± 0.03	0.36 ± 0.01
AKP (U/mg·protein)				
BBM	0.35 ± 0.00^b^	0.88 ± 0.12^a^	1.10 ± 0.17^a^	1.08 ± 0.11^a^
LAP (mU/mg·protein)				
BBM	1.88 ± 0.16^b^	4.54 ± 0.42^a^	3.55 ± 0.17^a^	3.30 ± 0.36^a^

*Abbreviations*. AKP, alkaline-phosphatase; LAP, leucine-aminopeptidase; PS, pancreatic segments; IS, intestinal segments; BBM, brush border membranes. ^1^Data are presented as means ± SEM. Means in each row sharing the same superscript letter or absence of superscripts are not significantly different decided by one-way ANOVA with post-hoc Tukey HSD test (*P*  > 0.05). SEM, standard error of means.

## Data Availability

The data gatherings created and/or diagnosed at the time of the present research are obtainable from the relative writer on feasible requirement.
